# Acculturation is associated with left ventricular mass in a multiethnic sample: the Multi-Ethnic Study of Atherosclerosis

**DOI:** 10.1186/s12872-015-0157-3

**Published:** 2015-12-03

**Authors:** Valery S. Effoe, Haiying Chen, Andrew Moran, Alain G. Bertoni, David A. Bluemke, Teresa Seeman, Christine Darwin, Karol E. Watson, Carlos J. Rodriguez

**Affiliations:** Division of Public Health Sciences, Wake Forest School of Medicine, Medical Center Blvd, Winston Salem, NC 27127 USA; Department of Medicine, Columbia University College of Physicians & Surgeons, New York, NY USA; National Institutes of Health/Clinical Center, Bethesda, MD USA; Division of Geriatrics, University of California at Los Angeles, Los Angeles, CA USA; University of California at Los Angeles Research Center, Los Angeles, CA USA; Division of Cardiology, University of California at Los Angeles School of Medicine, Los Angeles, CA USA

**Keywords:** Acculturation, Left ventricular mass index, Cardiovascular risk, Ethnic disparities

## Abstract

**Background:**

Acculturation involves stress-related processes and health behavioral changes, which may have an effect on left ventricular (LV) mass, a risk factor for cardiovascular disease (CVD). We examined the relationship between acculturation and LV mass in a multiethnic cohort of White, African-American, Hispanic and Chinese subjects.

**Methods:**

Cardiac magnetic resonance assessment was available for 5004 men and women, free of clinical CVD at baseline. Left ventricular mass index was evaluated as LV mass indexed by body surface area. Acculturation was characterized based on language spoken at home, place of birth and length of stay in the United States (U.S.), and a summary acculturation score ranging from 0 = least acculturated to 5 = most acculturated. Mean LV mass index adjusted for traditional CVD risk factors was compared across acculturation levels.

**Results:**

Unadjusted mean LV mass index was 78.0 ± 16.3 g/m^2^. In adjusted analyses, speaking exclusively English at home compared to non-English language was associated with higher LV mass index (81.3 ± 0.4 g/m^2^ vs 79.9 ± 0.5 g/m^2^, *p* = 0.02). Among foreign-born participants, having lived in the U.S. for ≥ 20 years compared to < 10 years was associated with greater LV mass index (81.6 ± 0.7 g/m^2^ vs 79.5 ± 1.1 g/m^2^, *p* = 0.02). Compared to those with the lowest acculturation score, those with the highest score had greater LV mass index (78.9 ± 1.1 g/m^2^ vs 81.1 ± 0.4 g/m^2^, *p* = 0.002). There was heterogeneity in which measure of acculturation was associated with LV mass index across ethnic groups.

**Conclusions:**

Greater acculturation is associated with increased LV mass index in this multiethnic cohort. Acculturation may involve stress-related processes as well as behavioral changes with a negative effect on cardiovascular health.

## Background

Increased left ventricular (LV) mass, or left ventricular hypertrophy (LVH) is an independent risk factor for cardiovascular disease events, and the prevalence of LVH varies between race/ethnic groups [1]. In a recent analysis of Hispanic, African-American, Chinese, and White participants in the Multi-Ethnic Study of Atherosclerosis (MESA), all Hispanic subgroups had a higher mean LV mass and a higher prevalence of LVH compared with White and Chinese participants at the time of the baseline study examination. Hypertension is strongly associated with the presence of LVH, but the race/ethnic differences in LV mass observed in MESA were not easily explained by a higher prevalence of hypertension among all of the Hispanic subgroups. In fact, Mexican-American participants had a higher mean LV mass and a higher prevalence of LVH compared with White and Chinese participants despite having a similar prevalence of hypertension and similar mean blood pressures [2].

Acculturation is the adoption of the traditions, values, attitudes and cultural practices of another country [3]. Acculturation may involve stress-related processes as well as behavioral changes. A number of studies have linked higher acculturation to a higher prevalence of hypertension [4–7]. Consistent with most, but not all prior studies, an analysis from MESA found that acculturation was associated with hypertension [5]. However, the association between acculturation parameters and hypertension within race/ethnic groups in this sample was not reported due to lack of statistical power. Given the strong association between hypertension and LVH, a positive association between greater acculturation and LVH would be expected. Acculturation may in part explain the relatively higher LV mass among Hispanic participants, when compared to other race/ethnic groups.

We used data from MESA to examine the associations between acculturation and LV mass. We hypothesized that a higher degree of acculturation, calculated using an acculturation score and acculturation characteristics: i) place of birth in or outside of the U.S.; ii) English vs. non-English language spoken at home; and iii) number of years living in the U.S. (in immigrants), would be associated with a higher mean LV mass, beyond risks accounted for by traditional risk factors for both acculturation and increased LV mass.

## Methods

### Study participants

Participants were drawn from MESA, a multi-center cohort study of the determinants of subclinical cardiovascular disease in 6814 men and women from four ethnic groups (non-Hispanic whites, Hispanics, African-Americans, and Chinese) aged 45–84 years. MESA cohort participants came from six US communities (Baltimore, MD; Chicago, Il; Forsyth County, NC; Los Angeles County, CA; Northern Manhattan, NY; and St. Paul, MN) and were free of any clinical cardiovascular disease at baseline. Details on the design and objectives of the MESA study have been previously published [8]. This study was approved by the Institutional Review Boards of each study site, and written informed consent was obtained from all participants.

The sample for the present analysis was 5004 men and women with complete baseline data on cardiac MRI assessment.

### Data collection and study variables

#### Measurement of left ventricular mass index

LV mass was measured using cardiac magnetic resonance (CMR) imaging technique. The MESA CMR protocol has been described and published elsewhere [9]. Imaging was performed using 1.5-Tesla MR scanners at each site using a standard protocol and read at a central site (Johns Hopkins University, Baltimore, MD). CMR was performed with a four-element, phased-array surface coil placed anteriorly and posteriorly, electrocardiogram gating, and brachial artery blood pressure monitoring. Cine images of the left ventricle were obtained with a temporal resolution of 50 milliseconds or less. LV mass was determined by the sum of the myocardial area (the difference between endocardial and epicardial contour) times slice thickness plus image gap in the end-diastolic phase multiplied by the specific gravity of myocardium (1.05 g/mL). LV mass was modeled as a continuous measurement, indexed by body surface area and expressed as g/m^2^.

#### Acculturation

Nativity, language spoken at home, and years living in the U.S. were used as proxy measures of acculturation. Nativity was categorized as U.S.-born and foreign-born (including those born in Puerto Rico). Language spoken at home was categorized as English, English and Chinese, English and Spanish, and non-English languages. Among foreign-born participants, number of years lived in the U.S. was categorized as less than 10 years, 10–19 years, and 20 or more years. These proxy measures were chosen for a number of reasons: they show strong correlations with existing acculturation scales, they explain much of the variance of existing scales, [10, 11] and they have also been widely used in other studies examining acculturation [5, 12–17].

For each participant we used the proxy measures of acculturation to compute an acculturation score. A score of 0–2 was assigned to language spoken at home (2 = English only; 1 = English and Chinese or English and Spanish; 0 = non-English languages). A score of 0–3 was assigned for years living in the U.S. combined with nativity (3 = U.S. born; 2 = foreign born and lived in the U.S. for 20 or more years; 1 = foreign born and lived in the U.S. for 10–19 years; 0 = foreign born and lived in the U.S. less than 10 years). For each participant, these individual scores were summed to obtain a summary acculturation score was calculated ranging from 0 (least acculturated) to 5 (most acculturated).

#### Covariates

Data used in this study were taken from the baseline examination (2000–2002) during which standardized questionnaires (administered in English, Spanish, or Chinese) and calibrated devices were used to obtain demographic data, smoking history, alcohol consumption, medical conditions, current prescription medication use, weight, and height. Three blood pressure readings were obtained with an appropriate-sized cuff at 1-min intervals with subjects seated after 5 min of rest using a Dinamap automated oscillometric sphygmomanometer (model Pro 100; Critikon, Tampa, FL). The average of the last two measurements was used for analysis. Hypertension was defined as a systolic blood pressure ≥140 mmHg or diastolic blood pressure ≥90 mmHg, use of blood pressure medicine or a self-report of hypertension. Fasting blood glucose was analyzed at a central laboratory. Diabetes was defined as a fasting blood glucose level of ≥126 mg/dl, use of hypoglycemic medications or insulin. Smoking use was defined as never, former, and current smokers. Alcohol consumption was defined as current drinkers or not. Annual income was categorized in 3 levels: participants earning < $20,000, $20,000 - $49,000, and > $49,000. Body mass index was calculated as weight (in kilograms) divided by the square of the height (in meters). Serum creatinine was measured on frozen serum specimens that were stored at −70 °C by rate reflectance spectrophotometry using thin film adaptation of the creatine amidinohydrolase method on the Vitros analyzer (Johnson & Johnson Clinical Diagnostics, Inc.). Physical activity was measured as the number of hours of exercise per week.

#### Statistical analysis

Sample characteristics by acculturation score or race/ethnicity were summarized using counts and percentages for categorical variables and mean with standard deviation for continuous variables. Linear regression models were used to examine the association between various acculturation factors and LVMI. We started with unadjusted models, and then proceeded to fit models adjusted for age, sex, serum creatinine, smoking status, income level, physical activity, diabetes status, and systolic blood pressure. Least square means (for categorical predictors) and beta coefficients (for continuous predictors) and associated standard errors are reported. A p-value of less than 0.05 was considered statistically significant. All analyses were performed using SAS 9.3 software (SAS Institute; Cary, NC).

## Results

### Cohort characteristics

The mean age of the sample was 62.1 ± 10.1 years, 39.1 % were white, 25.7 % African-American, 22.1 % were Hispanic, and 13.0 % Chinese. The mean LVMI of the entire cohort was 78.0 ± 16.3 g/m^2^. Compared to participants with lower acculturation scores, those with higher scores had higher mean systolic blood pressure and prevalence of hypertension, higher body mass index, tended to be current smokers and drinkers, and were more educated (Table 1). In contrast, a higher acculturation score was associated with a lower prevalence of diabetes. The annual income was $20,000 or less for half of participants who were least acculturated compared to $50,000 or more for half of those who were most acculturated.

**Table 1:** Baseline characteristics of study participants by acculturation score

Characteristic	Acculturation score = 0 *N* = 188	Acculturation score = 1 *N* = 260	Acculturation score = 2 *N* = 547	Acculturation score = 3 *N* = 169	Acculturation score = 4 *N* = 311	Acculturation score = 5 *N* = 3192
Age, years	60.7 ± 10.3	61.5 ± 10.9	62.1 ± 9.7	61.0 ± 10.4	62.2 ± 10.6	62.4 ± 10.0
Females, %	54.3	50.4	51.9	50.9	53.1	52.7
Educational level, %						
< High school	37.8	35.0	48.3	27.8	16.1	6.7
High school or college	55.9	55.0	44.4	55.0	64.6	69.9
> College	6.4	10.0	7.3	17.2	19.3	23.4
Annual income, %						
< $20,000	55.3	47.3	41.9	22.8	22.7	14.3
$20,000–$49,000	32.4	36.3	39.1	41.9	34.1	36.3
> $49,000	12.3	16.4	19.0	35.3	43.2	49.4
Diabetes, %	12.8	12.3	16.5	11.8	10.6	10.8
Hypertension, %	28.7	37.3	42.8	34.9	40.8	44.5
Smoker, %						
Never	68.6	70.4	66.9	58.6	48.2	44.8
Former	22.3	20.4	24.5	26.6	39.6	41.1
Current	9.0	9.2	8.6	14.8	12.2	14.1
Exercise, hrs/week	16.3 ± 26.1	18.3 ± 24.2	15.8 ± 21.9	26.2 ± 32.6	28.1 ± 37.9	30.2 ± 44.1
Current alcohol use, %	58.6	64.0	61.5	67.2	75.8	70.6
Systolic BP, mm Hg	124 ± 22	124 ± 22	127 ± 21	124 ± 21	125 ± 20	126 ± 21
Diastolic BP, mm Hg	71 ± 10	72 ± 10	72 ± 10	71 ± 11	71 ± 11	72 ± 10
Body mass index, kg/m^2^	25.3 ± 4.2	25.3 ± 4.3	27.1 ± 4.5	27.9 ± 5.0	28.4 ± 4.9	28.3 ± 5.0
Mean LVMI, g/m^2^	75.2 ± 14.3	75.8 ± 13.9	77.4 ± 15.5	78.5 ± 21.3	80.1 ± 16.7	78.2 ± 16.5

Data are mean ± SD for continuous variables or percentages for categorical variables

*BP* indicates blood pressure, *LVMI* left ventricular mass index

### LVMI and acculturation factors

In unadjusted analysis, among participants born out of the US, LVMI increased with increasing number of years lived in the US; those who had lived 20 years or more had a higher mean LVMI (78.3 ± 0.5 g/m^2^) compared to those who had lived for less than 10 years (LVMI 75.5 ± 1.1 g/m^2^, *p* = 0.01) (Table 2). LVMI in participants born in the U.S. and outside the U.S. was not different (*p* = 0.2).

**Table 2:** Unadjusted and adjusted mean left ventricular mass index by acculturation characteristic

Acculturation characteristic	Unadjusted analysis			Adjusted analysis^a^		
	Mean LVMI (g/m^2^)	SE	p-value	Mean LVMI (g/m^2^)	SE	p-value
Language						
Non-English spoken at home	76.8	0.5	0.01	79.9	0.5	0.02
Mixed languages at home	78.6	1.1		81.0	0.9	
English spoken at home	78.4	0.3		81.3	0.4	
Nativity + Years in the US						
Foreign born and in US for < 10 years	75.5	1.1	0.01	79.5	1.1	0.02
Foreign born and in US for 10–19 years	75.9	0.9		79.5	0.9	
Foreign born and in US for ≥ 20 years	78.3	0.5		81.6	0.7	

*LVMI* indicates left ventricular mass index, *SE* standard error

^a^Models adjusted for age, sex, income, serum creatinine, smoking status, physical activity, diabetes status, and systolic blood pressure

In multivariable analysis, after adjustment for age, sex, serum creatinine, smoking status, income level, physical activity, diabetes status, and systolic blood pressure, exclusively speaking English at home compared to non-English language was associated with higher LVMI (81.3 ± 0.4 g/m^2^ versus 79.9 ± 0.5 g/m^2^, *p* = 0.02) (Table 2). Among foreign-born participants, after adjustment, having lived in the U.S. for 20 years or more compared to having lived for less than 10 years was associated with greater LVMI (81.6 ± 0.7 g/m^2^ versus 79.5 ± 1.1 g/m^2^, *p* = 0.02).

### LVMI and acculturation score

In unadjusted analysis, increasing acculturation score was associated with greater mean LVMI (75.2 ± 1.2 g/m^2^ versus 78.2 ± 0.3 g/m^2^ for a score of 0 and 5, respectively, *p* < 0.01). This association persisted after adjustment (78.9 ± 1.1 g/m^2^ versus 81.1 ± 0.4 g/m^2^ for a score 0 and 5, respectively, *p* = 0.002) (Table 3).

**Table 3:** Adjusted mean left ventricular mass index by acculturation score

Acculturation score	Adjusted analysis		
	Mean LVMI (g/m^2^)	SE	p-value
Least acculturated (score = 0)	78.9	1.1	0.002
Acculturated (score = 1)	78.9	0.9	
Acculturated (score = 2)	80.5	0.7	
Acculturated (score = 3)	81.6	1.1	
Acculturated (score = 4)	83.4	0.8	
Most acculturated (score = 5)	81.1	0.4	

Models adjusted for age, sex, income, serum creatinine, smoking status, physical activity, diabetes status, and systolic blood pressure

*LVMI* indicates left ventricular mass index, *SE* standard error

### LVMI and acculturation by ethnicity

Blacks had the highest prevalence of hypertension (56.9 %), followed by Hispanics (39.6 %), Chinese (36.6 %), and Whites (36.3 %) (Table 4). Similarly, mean LVMI was highest for Blacks (81.3 ± 18.0 g/m^2^), followed by Hispanics (80.4 ± 16.6 g/m^2^), Whites (75.8 ± 15.2 g/m^2^), and Chinese (73.9 ± 13.6 g/m^2^). The majority of Chinese (87.2 %) and about half of Hispanics (51.2 %) had an acculturation score of 2 or less. Among Chinese (96.2 %) and Hispanics (66.4 %) born outside the U.S., a great majority had lived in the U.S. for 10 years or more (79.5 and 88.3 % respectively). Whites and Blacks had higher acculturation scores of 3 or more (99.9 and 99.7 %, respectively).

**Table 4:** Clinical and acculturation characteristics by ethnicity

Characteristic	Non-Hispanic White	Chinese	Black	Hispanic
N	1957	653	1288	1106
Diabetes, n (%)	113 (5.8)	81 (12.4)	215 (16.7)	172 (15.6)
Hypertension, n (%)	710 (36.3)	239 (36.6)	733 (56.9)	438 (39.6)
Systolic BP, mmHg	122 ± 20	123 ± 21	131 ± 21	126 ± 22
Diastolic BP, mmHg	70 ± 10	72 ± 10	75 ± 10	72 ± 10
Current smoker, n (%)	216 (11.1)	35 (5.4)	229 (17.9)	155 (14.0)
Exercise, hrs/week	1707 ± 2248	1145 ± 1472	1879 ± 3049	1361 ± 2009
Body mass index, kg/m^2^	27.3 ± 4.7	23.9 ± 3.3	29.4 ± 5.2	28.9 ± 4.5
Mean LVMI, g/m^2^	75.8 ± 15.2	73.9 ± 13.6	81.3 ± 18.0	80.4 ± 16.6
Percent life in the US	0.7 ± 0.3	0.3 ± 0.2	0.6 ± 0.3	0.5 ± 0.2
Non-English spoken at Home, n (%)	52 (2.7)	573 (87.8)	34 (2.6)	604 (54.6)
Born out of US, n (%)	136 (7.0)	630 (96.5)	143 (11.2)	764 (69.1)
Nativity + Years in the US, n (%)				
Foreign-born & in US for <10 years	5 (0.3)	120 (19.7)	5 (0.4)	79 (7.8)
Foreign-born & in US for 10–19 years	11 (0.6)	196 (32.2)	21 (1.7)	95 (9.3)
Foreign-born & in US for ≥ 20 years	90 (4.7)	269 (44.2)	84 (6.8)	501 (49.3)
US-born	1817 (94.5)	23 (3.8)	1135 (91.2)	342 (33.6)
Acculturation Score, n (%)				
Least acculturated (0)	0 (0)	115 (21.1)	0 (0)	73 (7.3)
Acculturated (1)	0 (0)	168 (30.8)	1 (0.1)	91 (9.1)
Acculturated (2)	1 (0.1)	192 (35.2)	3 (0.2)	351 (34.9)
Acculturated (3)	12 (0.6)	33 (6.1)	18 (1.5)	106 (10.5)
Acculturated (4)	71 (3.8)	21 (3.9)	77 (6.3)	142 (14.1)
Most Acculturated (5)	1803 (95.6)	16 (2.9)	1130 (91.9)	243 (24.2)

Data are mean ± SD for continuous variables or number (percentages) for categorical variables

*BP* indicates blood pressure, *LVMI* left ventricular mass index

Associations of acculturation with LVMI varied across ethnic groups. In Table 5, for Blacks, being born outside the U.S. compared to being born in the U.S. (85.8 ± 1.4 g/m^2^ versus 82.6 ± 0.7 g/m^2^, *p* = 0.03) was associated with higher mean LVMI after adjustment for age, sex, income level, serum creatinine, smoking, physical activity, diabetes, and systolic blood pressure. The association between acculturation score and LVMI also varied across ethnic groups. In Blacks, there appeared to be a significant non-linear association between acculturation score and LVMI. In Chinese participants, however, the results show a graded increase in LVMI across acculturation scores, though not significant.

**Table 5:** Adjusted mean left ventricular mass index by acculturation characteristic and by ethnicity

Acculturation characteristic	Race/Ethnicity							
	Non-Hispanic White		Chinese		African-American		Hispanic	
	LVMI, g/m^2^, mean ± SE	p-value	LVMI, g/m^2^, mean ± SE	p-value	LVMI, g/m^2^, mean ± SE	p-value	LVMI, g/m^2^, mean ± SE, p	p-value
Language								
Non-English spoken at home	80.2 ± 1.9	0.56	76.5 ± 0.9	0.24	83.4 ± 3.4	0.69	82.8 ± 0.8	0.075
Mixed languages at home	78.3 ± 4.9		78.2 ± 1.9		78.9 ± 4.7		82.3 ± 1.2	
English spoken at home	78.2 ± 0.7		79.6 ± 2.1		82.9 ± 0.7		84.9 ± 0.9	
Nativity								
US-born	78.2 ± 0.7	0.19	76.0 ± 2.6	0.74	82.6 ± 0.7	0.027*	84.2 ± 0.9	0.24
Foreign-born	79.7 ± 1.3		76.9 ± 0.9		85.8 ± 1.4		83.0 ± 0.8	
Nativity + Years in US								
Foreign born - in US for < 10 years	90.2 ± 6.2	0.92	75.9 ± 1.4	0.37	80.5 ± 10.7	0.73	84.3 ± 1.9	0.61
Foreign born - in US for 10–19 years	87.8 ± 4.7		76.7 ± 1.2		86.7 ± 5.9		82.4 ± 1.7	
Foreign born - in US for ≥ 20 years	87.6 ± 2.7		77.7 ± 1.1		88.5 ± 3.9		82.6 ± 0.9	
Acculturation score								
Least acculturated (0)	-	0.72	75.1 ± 1.5	0.32	-	0.024*	84.3 ± 1.9	0.25
Acculturated (1)	-		76.2 ± 1.2		-		83.1 ± 1.7	
Acculturated (2)	84.5 ± 12.8		76.8 ± 1.2		80.15 ± 8.9		82.9 ± 0.9	
Acculturated (3)	78.9 ± 3.7		77.3 ± 2.3		88.7 ± 3.7		82.7 ± 1.5	
Acculturated (4)	79.4 ± 1.7		81.9 ± 2.8		87.8 ± 1.9		82.5 ± 1.3	
Most Acculturated (5)	77.8 ± 0.7		76.2 ± 3.2		82.8 ± 0.7		85.5 ± 1.0	

Adjusted for age, sex, income, serum creatinine, smoking status, physical activity, diabetes status, and systolic blood pressure

*LVMI* indicates left ventricular mass index, *SE* standard error

*p < 0.05

To assess if indexing LV mass to body surface area accounted for the effects of obesity on the association between LV mass and measures of acculturation, we performed sensitivity analyses using LV mass as the outcome variable. The multivariable models were adjusted for the same covariates described above and body mass index. The association between LV mass and the different measures of acculturation (language, nativity, and number of years in the U.S.) were significant and similar to those described above.

## Discussion

This study examined the association between acculturation and LVMI in a multi-ethnic cohort of individuals aged 45–84 years at baseline and who had no discernable clinical cardiovascular disease. From the findings, higher levels of acculturation are associated with increased LVMI. Also, LVMI varied by language spoken at home and time spent in the U.S. but not by nativity; different measures of acculturation appeared to have varying effects in differences race/ethnic groups, highlighting the complexity of the acculturation concept.

Among foreign-born participants, having lived in the U.S. for longer periods of time was associated with a higher mean LVMI, even after adjusting for traditional CVD risk factors. In fact mean LVMI increased after 20 or more years of residence in the U.S., compared to those who had lived in the U.S. for less than 20 years. Evidence suggests that the health advantage exhibited by foreign-born individuals over U.S.-born individuals tends to decrease with duration of stay in the U.S. [18, 19]. This decline in the health with increased duration of stay in the U.S. has also been reported for other health measures like obesity [16, 19–21] and heart disease [22]. One explanation could be that some immigrant groups (non-U.S. Whites, Hispanics, and Asians) may be less likely, than U.S.-born individuals, to discuss dietary or physical activity measures with their clinicians, [19] probably in part due to patient-provider characteristics which affect care such as language barriers and cultural sensitivity.

Participants who spoke exclusively English at home had higher LVMI compared to those who spoke a language other than English. This finding may be explained by the fact that those who spoke exclusively English may have been either those born in the U.S. or those who had lived in the U.S. for longer periods of time. Ninety-seven percent of Blacks (91 % born in the U.S.) and 97 % of non-Hispanic Whites (94 % born in the U.S.) spoke exclusively English at home. These may have been participants with higher acculturation levels (96 % of non-Hispanic whites and 92 % of Blacks had an acculturation score of 5). This finding corroborates with an analysis using the same cohort which showed a higher prevalence of hypertension, a strong predictor of LVMI, among those who spoke English at home [5]. Other studies have described different associations between language spoken and hypertension. In one study, those who spoke Russian at home reported a higher prevalence of hypertension than those who spoke English [23]. This was attributed to a higher baseline prevalence of self-reported hypertension among those born in Eastern/Central Europe, than US-born whites.

In our study, the association between acculturation and LVMI did not differ when birthplace alone was considered (U.S.-born vs. foreign-born). This finding may be attributed to the fact that among participants born out of the U.S. (33 % of sample), the majority (76 %) had lived in the U.S. for at least 10 years and thus may have had acculturation levels comparable to those born in the U.S.

The race/ethnic stratified analysis demonstrates the complexity and heterogeneity of the associations of acculturation measures and LVMI among the different immigrant groups. Overall, blacks had higher mean LVMI than other race/ethnic groups. This risk was even higher among foreign-born than U.S.-born blacks. The increased risk in blacks may be due to a number of factors including psychosocial stress, chronic adrenergic stimulation [24, 25] and increased sodium retention, [26] both of which are disproportionately increased in blacks. An association between these factors and increased LVMI has been described in other studies [27, 28]. Place of birth, and not language spoken at home, may have been an important dimension of acculturation among blacks in the US (who may be from Haiti, the Caribbean, or Africa) since many countries in the Caribbean and in Africa already have English as the national language. Among Hispanics, preferential English speaking at home (greater acculturation) was associated with increased LVMI. There is strong evidence that points towards a negative effect of greater acculturation and health behaviors, including diet, illicit drug, alcohol and tobacco use, [29] all of which are associated with increased LVMI [30–32].

Despite having higher levels of physical activity, the most acculturated participants had higher mean LVMI. Similarly, blacks with the highest total number of hours of exercise per week had the highest mean LVMI of all ethnicities; Chinese with the least total number of hours of exercise had the least mean LVMI. The fact that the more acculturated participants were more physically active despite having higher LVMI may be explained by a number of factors. First, physical activity may not be an important factor contributing to decreased LVMI in our sample. This is consistent with results from one study which showed that fat mass, rather than inactivity, is an important contributor to disease risk in young Mexican and Mexican–American women [33]. Second, our measure of physical activity (hours of exercise per week) may not be a good correlate of the effects of exercise on LVMI.

To the best of our knowledge, this is the first study investigating the association between acculturation and LVMI. The multiethnic nature of our sample makes it possible to compare the independent associations of measures of acculturation among the different race/ethnic groups. However, the cross-sectional nature of our analysis makes it impossible to draw any inferences on a causal link between acculturation and LVMI. Another limitation of our study is the scope of our measure of acculturation which may have influenced some of our non-significant findings. Several studies have used different surrogates for acculturation, and our measure may not fully cover the spectrum of acculturation and its related cardiovascular health effects. It is therefore important to consider the measures of acculturation used and outcomes under study when comparing our findings with that of other studies. Also, due to sample size limitations of our race/ethnic stratified analyses, we may have had insufficient power to detect the presence of other associations between measures of acculturation and LVMI among race/ethnic groups. Finally, residual confounding via measurement error may possibly explain some of the associations found, although we would expect significant associations given the results of other studies examining acculturation and other health effects. Our study nevertheless found significant associations between acculturation measures and increased LVMI.

Regardless of the process of acculturation, lifestyle modification (via physical activity, diet, and smoking cessation) provides cardiovascular health benefits. The present study, however, identifies a group a group of individuals (more acculturated) which is at risk of developing increased LVM, and consequently CVD. This reinforces the notion that the immigrant process and making decisions on retaining one’s native culture while adapting to a new culture may exert a remarkable stress on cardiovascular health behaviors and subsequent health risks in certain individuals due to factors such as lack of healthcare access and social marginalization which will impede healthy lifestyle modifications.

## Conclusion

Given the growing size of the immigrant population in the U.S., it is important to study disease prevalence and associated risks in the different race/ethnic groups, in order to better tailor preventive strategies. Our study showed that different acculturation measures (language spoken at home, nativity, and length of stay in the U.S.) are important determinants of subclinical cardiovascular disease, and the study also highlights the heterogeneity and complexity of studying the acculturation process among different race/ethnic groups.
